# Chronic vs acute manipulations reveal degeneracy in a thermosensory neuron network

**DOI:** 10.17912/micropub.biology.000355

**Published:** 2021-01-15

**Authors:** Jihye Yeon, Asuka Takeishi, Piali Sengupta

**Affiliations:** 1 Department of Biology, Brandeis University, Waltham, MA; 2 Department of Biology, Brandeis University, Waltham, MA; 3 RIKEN Hakubi Research Team, RIKEN Cluster for Pioneering Research, RIKEN Center for Brain Science, Wako, Japan

## Abstract

Degenerate networks can drive similar circuit outputs. Via acute manipulation of individual neurons, we previously identified circuit components that are necessary and sufficient to drive starvation-dependent plasticity in *C. elegans* thermotaxis behavior. Here we find that when these components are instead silenced chronically, degenerate mechanisms compensate to drive this behavior. Our results indicate that degeneracy in neuronal network function can be revealed under specific experimental conditions.

**Figure 1. Chronic but not acute silencing of neurons identifies a degenerate thermosensory behavioral circuit:**
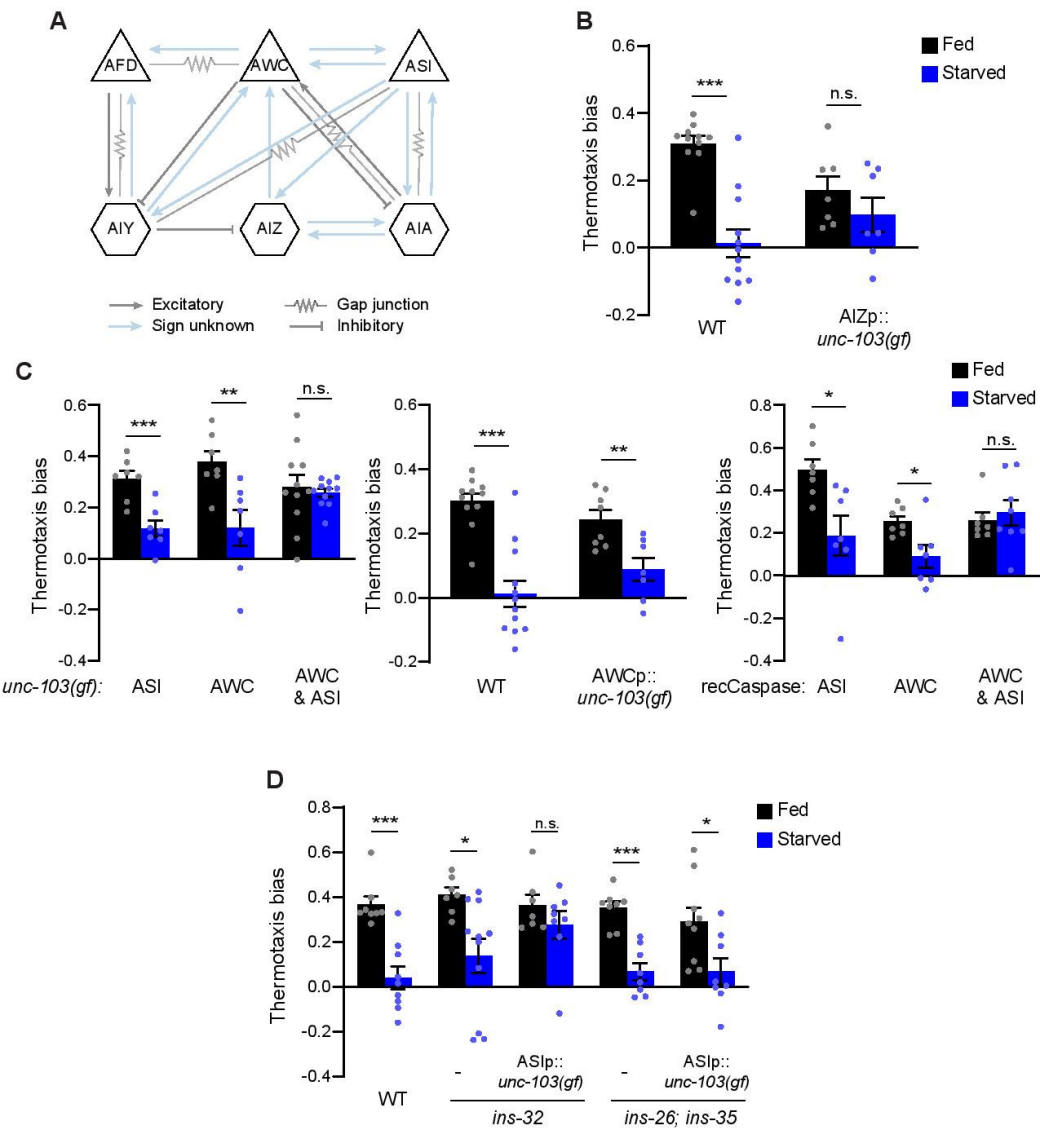
**A)** Simplified schematic of the connectivity of sensory neurons and interneurons discussed in this work. Adapted from (Cook *et al.*, 2019; White *et al.*, 1986) (www.wormwiring.org). **B)** Mean thermotaxis bias of fed and starved wild-type and transgenic animals expressing *unc-103(gf)* in AIZ. *unc-103(gf)* sequences were expressed using the same sequences and strategy used to drive HisCl1 expression to acutely silence AIZ (Takeishi *et al.*, 2020) (see Tables 1 and 2). Thermotaxis bias was calculated as [(run duration toward colder side – run duration toward warmer side)/total run duration]. **C)** Mean thermotaxis bias of fed and starved wild-type and transgenic animals expressing *unc-103(gf)* (left and center), and recCaspases (right). Regulatory sequences used to drive expression of transgenes in AWC and ASI were: (Left) AWC: *ceh-36del*, ASI: *srg-47*; (Center) AWC: *odr-1*; (Right) AWC: *ceh-36del,* ASI: *gpa-4* and *gcy-27* (also see Tables 1 and 2). **D)** Mean thermotaxis bias of fed and starved animals of the indicated genotypes. Alleles used were *ins-26(tm1983)*, *ins-32 (tm6109)*, and *ins-35(ok3297)*. The *srg-47* promoter was used to drive *unc-103(gf)* in ASI. In each graph, a dot represents the thermotaxis bias of a biologically independent assay comprised of 15 animals. Errors are SEM. *, ** and *** indicate different from fed at p<0.05, p<0.01, and p<0.001, respectively (Student’s t-test). n.s. not significant.

## Description

Neuronal networks can achieve similar outputs via distinct underlying circuit mechanisms (Beverly *et al.*, 2011; Marder *et al.*, 2015; Saideman *et al.*, 2007; Trojanowski *et al.*, 2014; Wang *et al.*, 2019). This degeneracy allows networks to maintain robustness without compromising functional flexibility (Cropper *et al.*, 2016; Edelman and Gally, 2001). Since the contribution of degenerate neuronal pathways is likely to be revealed under defined genetic or environmental conditions, it is challenging to identify and describe the contributions of such pathways to neuronal circuit function.

*C. elegans* exhibits experience-dependent thermotaxis behaviors (Hedgecock and Russell, 1975). When animals are placed on a thermal gradient at temperatures warmer than the temperature at which they were cultivated with bacterial food (cultivation temperature – *T_c_*), they move towards cooler temperatures (negative thermotaxis – NT). Genetic ablation experiments established that in well-fed animals, the AFD, AWC and ASI sensory neuron pairs act degenerately to drive NT under specific assay conditions (Beverly *et al.*, 2011; Ikeda *et al.*, 2020; Kuhara *et al.*, 2008; Mori and Ohshima, 1995). Starvation for 2-3 hrs prior to the assay disrupts NT, resulting in atactic behavior (Chi *et al.*, 2007; Hedgecock and Russell, 1975; Kodama *et al.*, 2006) (referred to here as ‘starvation-dependent plasticity’). Via acute chemogenetic and optogenetic manipulation of neuron activity during the behavioral assay (henceforth referred to as ‘acute silencing’), we recently showed that increased and decreased temperature responses in AWC and their postsynaptic AIA interneuron partners (Figure 1A), respectively, are necessary and sufficient for starvation-dependent plasticity in NT (Takeishi *et al.*, 2020). In the course of these experiments, we noted that while temperature responses in the AIZ interneurons (Figure 1A) were also modulated by starvation in a manner similar to responses in AIA, acute chemogenetic manipulation of AIZ activity had no effect on NT regardless of feeding conditions (Takeishi *et al.*, 2020). We speculated that AIZ may be a component of a degenerate thermotaxis behavioral circuit that contributes to starvation-dependent plasticity in NT under specific conditions.

We tested whether silencing AIZ throughout postembryonic development (henceforth referred to as ‘chronic silencing’) may reveal a role for this interneuron type in modulating thermotaxis. While histamine-HisCl1-mediated acute silencing of AIZ had no effect on NT (Takeishi *et al.*, 2020), chronic silencing via expression of an activated UNC-103 potassium channel [*unc-103(gf)*] (Reiner *et al.*, 2006) in this neuron significantly decreased NT regardless of feeding conditions (Figure 1B), possibly due to disruption of AIZ-mediated regulation of reversals and turns. In contrast, both acute and chronic silencing of AIA abolished NT similarly in fed and starved animals (Takeishi *et al.*, 2020). These results indicate that while AIA is able to drive NT when AIZ is acutely silenced, AIA is unable to compensate when AIZ is silenced chronically.

We next examined whether the contributions of sensory neurons upstream of AIA and AIZ (Figure 1A) to NT are also distinct based on the duration and/or timing of neuronal silencing. Acute chemogenetic or optogenetic silencing of AWC alone during thermotaxis abolished starvation-dependent plasticity in NT without affecting NT in fed animals (Takeishi *et al.*, 2020). Unexpectedly, we found that starved animals in which AWC has been chronically silenced via expression of *unc-103(gf)* orgenetic ablation (Beverly *et al.*, 2011; Chelur and Chalfie, 2007), continued to exhibit starvation-dependent plasticity in NT similar to the behavioral phenotype of wild-type animals (Figure 1C), but in stark contrast to the behavior of animals in which AWC is acutely silenced (Takeishi *et al.*, 2020). To confirm that the observed behavioral differences were not simply due to differences in the promoters used for chronic and acute silencing (see Methods), we also expressed *unc-103(gf)* under *odr-1* regulatory sequences; *odr-1* promoter sequences were used previously to drive HisCl1 and GtACR2 expression in AWC (Takeishi *et al.*, 2020). Animals expressing *odr-1*p*::unc-103(gf)* again exhibited starvation-dependent plasticity in NT (Figure 1C). The NT behavioral phenotypes of animals in which ASI was acutely or chronically silenced resembled those of wild-type animals (Figure 1C) (Takeishi *et al.*, 2020). We found that chronic silencing of both AWC and ASI together was necessary to fully abolish starvation-dependent plasticity in NT (Figure 1C), phenocopying the effects of silencing AWC alone acutely (Takeishi *et al.*, 2020). These results indicate that ASI can contribute to starvation-dependent plasticity in NT, but that a role for this neuron is only revealed when AWC is chronically but not acutely silenced.

We previously noted that in contrast to the lack of an effect of chronic silencing of AWC alone on starvation-dependent plasticity in NT, chronically blocking glutamatergic transmission from AWC was sufficient to abolish this plasticity, similar to the effects observed upon acutely silencing AWC alone (Takeishi *et al.*, 2020). A simple model to account for these seemingly contradictory observations is that AWC also releases a peptidergic signal upon starvation. Absence of this signal due to chronic silencing of AWC, but not upon acute AWC silencing or upon loss of glutamatergic signaling from AWC, may trigger ASI-dependent compensatory mechanisms. This model predicts that a loss-of-function mutation in such a peptide-encoding gene would phenocopy the effects of AWC chronic silencing on starvation-dependent plasticity in NT.

We reported a subset of insulin-like peptide (ILP) genes expressed in AWC which plays a role in transmitting information about food availability to influence the dauer developmental decision (Neal *et al.*, 2015). We tested whether one or more of these ILP genes may play a role in starvation-dependent plasticity in NT. The NT behavioral phenotypes of *ins-32* mutants alone or *ins-26; ins-35* double mutants were similar to those of wild-type animals in both fed and starved conditions (Figure 1D). However, while the NT behavioral phenotype of *ins-26; ins-35* mutants was not further altered upon ASI chronic silencing, chronic silencing of ASI in *ins-32* mutants abolished starvation-dependent plasticity in NT (Figure 1D). Thus, in this context, loss-of-function mutations in *ins-32* appear to phenocopy the effects of chronic AWC silencing.

We propose the following model to account for observations reported here and in our previous work (Takeishi *et al.*, 2020). Long-term chronic silencing of AWC may promote compensatory mechanisms via ASI to mediate starvation-dependent plasticity in NT. This compensation may be triggered by the prolonged absence of *ins-32* ILP signaling from AWC. Although the nature of the proposed ASI-dependent compensatory pathway is currently unclear, altered peptidergic signaling from ASI may play a role (Chen *et al.*, 2013). Acute AWC silencing during the behavioral assay alone may not be sufficient to induce this ASI-dependent compensatory mechanism. Alternatively, the relevant compensatory mechanism may be absent in adult animals. Developmental compensation in *C. elegans* neuronal networks has previously been reported and has been suggested to potentially be mediated via rewiring of synaptic connections or altered functions of other circuit components (Beverly *et al.*, 2011; Trojanowski *et al.*, 2014; White *et al.*, 2007). Rewiring may account for the differential requirements of AIA and AIZ in driving NT upon chronic and acute silencing. However, given the extensive peptidergic and hormonal signaling network in *C. elegans*, we instead favor the hypothesis that this compensation and degeneracy instead arises from reconfiguration of the ‘wireless’ connectome (Bargmann and Marder, 2013; Bentley *et al.*, 2016). Our results further reinforce that conclusions drawn about neuron circuit components driving specific behaviors should be interpreted with caution in the context of the specific manipulations performed.

## Methods

***C. elegans* growth**

*C. elegans* was maintained at 20°C with *E. coli* OP50 as the food source. To generate transgenic animals, 5-30 ng/μl of experimental plasmids were injected with co-injection markers (*unc-122*p*::gfp, unc-122*p::*dsRed*, *elt-7*p*::gfp,* or *elt-7*p*::mCherry*). Mutations were confirmed by sequencing. Strains used in this work are listed in Table 1.

**Molecular biology**

Promoters used in this work were (upstream of ATG): *odr-1* (AWC: 1.0 kb), *ceh-36del*p (AWC: 627 bp), *srg-47* (ASI: 650 bp), *odr-2b(3a)* (AIZ and others: 448 bp), *ser-2(2)* (AIZ and others: 4.7 kb). Promoters and cDNA sequences were cloned into the pPD95.77 expression vector. All plasmids were confirmed by sequencing. Plasmids used in this work are listed in Table 2.

**Thermotaxis behavioral assay**

Negative thermotaxis behavioral assays were performed as previously described (Takeishi *et al.*, 2020). Starved worms were obtained by transferring animals onto unseeded NGM plates for 3 hrs prior to the start of the assay. Assays were performed on a thermal gradient ranging from 23°C-28°C at 0.5°C/cm.

## Reagents

**Table:** 

**Table 1.** Strains used in this work.		
**Strain**	**Genotype**	**Source**
Wild type	N2 (Bristol)	CGC
PY12216	*oyEx676* [*ser-2(2)*p::*FRT*::*STOP*::*FRT*::*unc-103(gf)*::*SL2*::*gfp* + *odr-2b(3a)*p::*nFLP* + *elt-7*p::*gfp*]	This work
PY12220	*oyIs92* [*ceh-36del*p::*unc-103(gf)*::*SL2*::*mCherry* + *unc-122*p::*gfp*]	This work
PY7502	*oyIs85* [*ceh-36del*p::*TU813* + *ceh-36del*p::*TU814 +* *unc-122*p::*dsRed* + *srtx-1*p::*gfp*]	(Beverly *et al.*, 2011)
PY12221	*oyIs93* [*srg-47*p::*unc-103(gf)*::*SL2*::*mCherry* + *elt-7*p::*mCherry*]	This work
PY7505	*oyIs84* [*gpa-4*p::*TU813* + *gcy-27*p::*TU814 +* *gcy-27*p::*gfp* + *unc-122*p::*dsRed]*	(Beverly *et al.*, 2011)
PY7507	*oyIs84* [*gpa-4*p::*TU813* + *gcy-27*p::*TU814 +* *gcy-27*p::*gfp* + *unc-122*p::*dsRed]; oyIs85*[*ceh-36del*p::*TU813* + *ceh-36del*p::*TU814 +* *unc-122*p::*dsRed* + *srtx-1*p::*gfp*]	(Beverly *et al.*, 2011)
PY12223	*oyIs93* [*srg-47*p::*unc-103(gf)*::*SL2*::*mCherry* + *elt-7*p::*mCherry*]; *oyIs92* [*ceh-36del*p::*unc-103(gf)*::*SL2*::*mCherry* + *unc-122*p::*gfp]*	This work
PY12217	*oyEx677* [*odr-1*p::*unc-103(gf)*::*SL2*::*mCherry* + *unc-122*p::*gfp]*	This work
PY10712	*ins-26(tm1983); ins-35(ok3297)*	(Neal *et al.*, 2015)
PY12219	*ins-26(tm1983); ins-35(ok3297); oyIs*93 [*srg-47*p::*unc-103(gf)*::*SL2*::*mCherry* + *elt-7*p::*mCherry*]	This work
PY10808	*ins-32 (tm6109)*	(Neal *et al.*, 2015)
PY12218	*ins-32 (tm6109)*; *oyIs93*[*srg-47*p::*unc-103(gf)*::*SL2*::*mCherry* + *elt-7*p::*mCherry*]	This work
		
**Table 2.** List of plasmids used in this work.		
**Plasmid**	**Description**	**Source**
PSAB1255	*ser-2(2)*p::*FRT*::*STOP*::*FRT*::*unc-103(gf)*::*SL2*::*gfp*	This work
PSAB1253	*ceh-36del*p::*unc-103(gf)*::*SL2*::*mCherry*	This work
PSAB1254	*srg-47*p::*unc-103(gf)*::*SL2*::*mCherry*	This work
PSAB1256	*odr-1*p::*unc-103(gf)*::*SL2*::*gfp*	This work
pWY046	*odr-2b(3a)*p::*nFLP*	(Takeishi *et al.*, 2020)
